# Assessment of Healthcare Waste Management Paradigms and Its Suitable Treatment Alternative: A Case Study

**DOI:** 10.1155/2018/6879751

**Published:** 2018-07-29

**Authors:** Mohammad Mehedi Hasan, M. Habibur Rahman

**Affiliations:** ^1^Hydraulic Research Directorate, River Research Institute (RRI), Faridpur 7800, Bangladesh; ^2^Department of Civil Engineering, Bangladesh University of Engineering and Technology (BUET), Dhaka 1000, Bangladesh

## Abstract

The management and treatment of healthcare waste (HCW) are of great concern owing to its potential hazard to human health and the environment, particularly in developing countries. Nowadays, various technological alternatives are gaining momentum as efficient and favorable waste management options across the world. However, selecting a suitable technology as well as an effective waste management approach for the treatment of HCW is still a challenging task for the municipal authorities. This study renders a comprehensive analysis of healthcare waste management (HCWM) practices and the technological options for its better management through a case study in Khulna, the southwestern division of Bangladesh. A number of healthcare establishments (HCEs) in the study area were selected and a questionnaire survey, as well as field investigations, was performed to find out the present status of HCWM and its limitations. An assessment of different technological alternatives was also carried out using Sustainability Assessment of Technologies (SAT) methodology which could pave the way for treating hazardous waste more efficiently in the Khulna metropolitan area. The study revealed that the overall HCW generation rate and hazardous HCW generation rate in Khulna city were 0.90 kg bed^−1^ day^−1^ and 0.18 kg bed^−1^ day^−1^, respectively. Assessment of management system revealed that 56% (*n* = 38) of workers did not receive any form of training in the handling of hazardous waste. Around 54% (*n* = 47) of them did not use any safety equipment or clothing. It has been found from the study that, among different technological alternatives based on the final score, incineration was the most suitable option for the treatment of hazardous waste in Khulna. Finally, some guidelines have been put forward to improve its existing management practices.

## 1. Introduction

Healthcare waste has been a growing concern across the world over the last few years [1]. HCW is defined as all types of waste generated from HCEs, whether it is infectious or noninfectious in nature, chemicals, and hazardous as well as nonhazardous materials [2]. In developing countries, HCW poses a serious threat due to its potential for causing environmental and public health hazards. A lack of awareness among health professionals as well as general population regarding improper handling of HCW, the absence of an effective regulatory framework and national policy, and financial strains are the major impediments of adequate HCWM, and all increase the potential risk of environment and public health hazards. Bangladesh, a developing country, also experiences the same obstructions, which exerts a tremendous impact on the environment and public health. Mushrooming growth of healthcare facilities (HCFs) in urban areas accentuates the problem to a large extent. About 85% of the waste generated by healthcare activities is general, nonhazardous in nature and the remaining 15% is considered hazardous materials which may be infectious, toxic, or radioactive [3]. This small portion of HCW may pose various environmental and health risks if not managed or disposed of properly. For the treatment and disposal of HCW, a range of technologies have been developed so far. However, identification of an appropriate waste treatment technology for the selected site is still a challenging task for the planners and decision-makers, especially in developing countries. To this end, a number of factors are involved. These include the quantification and characterization of local waste, degrees of safety, technological suitability, and the cost and impact on the environment.

Khulna, the third largest metropolitan city of Bangladesh, is presently encountering the impacts of inadequate management of HCW [4]. In many cases in these cities, most of the hospitals and clinics, in both the public and private sectors, have either a nonexistent or an outdated HCWM system. Wastes produced from the HCEs in the city are not treated or smashed properly. Instead, they are thrown into dustbins, consequently causing health hazards. These waste products mingled with general solid waste from different households contaminated the air and water as well as the wider environment. Moreover, bacteria such as* Pseudomonas*,* Staphylococcus*,* Streptococcus,* and so on are produced from these products [5]. Infections which may result from these bacteria include meningitis, AIDS, hemorrhagic, diarrhea, tuberculosis, and various skin diseases [6]. Comprehending the intensity of the problem, a nongovernmental organization (NGO) has already extended its assistance to Khulna City Corporation (KCC) for better management of HCW. However, the present management system is dangerous to the environment and human health [4]. HCWM is one of the most ignored parts of the white-collar process in Bangladesh. Improper management of HCW exposes health workers, waste handlers, and the community to infections, toxic effects, and a higher potential risk of injury [7]. It was observed that consciousness regarding health hazards of HCW among professional as well as general people is very low. With the rapid advancement of urbanization, the environment as well as the health conditions of city dwellers will be an unpleasant one.

Considering the consequences of environmental and potential health risks, a number of studies have investigated various aspects of waste management in Khulna city. Rahman et al. [8] analyzed the suitability of solid waste disposal sites using a GIS approach in Khulna city. Moniruzzaman et al. [9] disclosed the recycling practices of solid waste in Khulna city. Bari et al. [10] revealed the scenario of solid waste reuse in Khulna city. Another study conducted by Ahsan et al. [11] evaluated the role of private organizations in waste management. Therefore, most of the studies in this area have been focused on solid waste management. However, much less attention has been paid to HCWM issues specifically, although, HCW pose a significant threat to the environment and public health in Khulna [4]. This study was conducted to investigate the existing HCWM patterns and their downsides in the KCC area. An effort was also made to explore the possibility of developing an efficient waste management system for the metropolis. Therefore, an assessment of the different technological alternatives was also accomplished using SAT methodology in order to comprehend their applicability in Khulna for the betterment of the present situation. For the technology selection in the environmental arena, various decision-making tools and approaches have been developed including AHP, Matrix method, ANP, Fuzzy, and VIKOR where each method has its own criteria in finding the best alternatives. SAT is an apposite approach for incorporating technical, environmental, economic, and social considerations with the primary emphasis on developmental aspects and environmental issues [12]. Examples of investigations carried out using SAT methodology includes those of Suthapanich [13] and Rafiee et al. [14], where assessment and selection of best waste treatment technologies were explored. Furthermore, quantification and analysis of the physical composition of HCW were performed, since quantitative assessment of HCW generation is usually the main basis for any waste management plan [15].

## 2. An Overview of Various HCW Treatment Technologies

The HCW treatment options include incineration, microwaving, autoclaving, hydropulping, and compaction. Incineration is a perfect method for all types of HCW, both hazardous and nonhazardous. Combustion temperature is more than 1800°F [16]. All hazardous and toxic elements can be destroyed effectively. The method significantly reduces the volume of waste by up to 95% of its original volume [17, 18] which is highest among all the methods. However, investment cost for incineration is very high and it may emit unwanted pollutants. Conversely, microwaving has less impact on the environment compared to incineration because there are no combustion emissions produced by the system. It can reduce the waste volume by approximately 80%. Microwaving is not suitable for pathological waste and requires a strict monitoring system [17, 19].

Autoclaving is suitable for sterilizing hazardous waste. However, the volume of waste cannot be reduced in this process. It is not suitable for recognized body parts [19]. On the other hand, the hydropulping, an oxidation technique, has the required water content of approximately 80% as a result of an increase in weight; however, the resultant volume can be as little as 30% of the original volume. The use of a pulping system is highly controversial for clinical waste treatment [18]. The compaction system can reduce the waste volume up to 60%. However, sterilization is not possible in this method [17]. A thorough description of technology with their strengths and weaknesses, as well as some key factors for technology selection, has been presented in preceding publications [20–22].

## 3. Sustainability Assessment of Technologies (SAT) Methodology in Brief [12]

The SAT methodology was introduced by the International Environmental Technology Centre (IETC) of the United Nations Environment Program (UNEP). Sustainability is a major concern in this methodology which incorporates technical suitability, environmental aspects, social acceptability, and economic feasibility. The SAT methodology addresses first strategic and then, operational level assessments.

*Strategic-Level Assessment. *A situational analysis is undertaken by planners, elected representatives, and decision-makers in this level of assessment encompassing the collection of baseline data, preliminary information, relevant stakeholder consultations, and mapping. Afterward, the measurable target is defined for a particular issue selected in the course of stakeholder discussions. A “target” specifies by what means an identified single issue can be mollified.

*Operational Level Assessment. *The methodology continues to an operational level assessment next to the macrolevel or strategic-level assessment, in which technical staff, experts, engineers, and so on assess available technology options.

The subsequent three-phased approach is used in this level of assessment.

*Phase 1: Screening. *Available technological options are scrutinized against finalized technical and environmental criteria which are usually in the form of logical operators (i.e., Yes/No types). Scrutinizing can be performed by a suitable stakeholder group with or without the help of expert opinion.

*Phase 2: Scoping. *Eligible technological alternatives from the preceding step are then subjected to go through the extensive scoping step. Scoping criteria may be developed under four broad categories including technical, environmental, economic, and social aspects. The weightage for each criterion is ascribed based on how the stakeholders gave an importance on it. The score was assigned on the scale of 1 to 10 in the descending order. It is to be noted that several quantification techniques can be applied in the scoping phase based on the intricacy as well as sensitivity of the decision to be made. Moreover, competency and the capabilities of stakeholder groups can influence the choice of aggregation method. The weighted sum matrix method is likely said to be the simplest one.

*Phase 3: Detail Assessment. *Technological options with best overall scores from the scoping phase are then subjected to further methodical assessment in this phase. This assessment is situation-specific and requires comprehensive and quantitative information for each criteria topic to assist decision-making. At this stage, a composite star diagram can be used to summarize and present data about various traits, fact related to each topic.

The steps, criteria, and indicators defined in SAT methodology should be followed as a general guide. They should be revised and adapted through consultative meetings to meet local conditions. An inclusive interpretation of SAT methodology can be obtained from UNEP [12].

## 4. Methodology

### 4.1. Study Site

Khulna, one of the major divisions in Bangladesh with an area of about 60 km^2^, stands on the banks of River Rupsha and Bhairab. It is located in the southwestern part of the country at 22°48′′N to 22°54′′N Latitude and 89°31′′E to 89°34′′E Longitude. The present population of Khulna city is about 0.7 million. The city has experienced a high rate of population growth in the last few decades principally due to migration from the adjacent city as it is the administrative headquarters of Khulna division and the regional center for higher education, better treatment facilities, business, and so on. To meet the demand of its large and ever-increasing population, a good number of HCFs have been developed in the city. The nearby small districts also depend on Khulna for better treatment facilities. This regional importance has also accelerated the growth of the health sector in Khulna metropolis.

### 4.2. Study Design

This study is a cross-sectional survey and of explorative nature. HCEs in the KCC area that allowed us to collect relevant HCW data were selected for this study. Finally, 20 different HCEs (Table 1), including diagnostic centers residing in KCC area, were selected to carry out this investigation. The purpose of such selection was to obtain representative features of the existing HCWM status of Khulna municipality as well as to determine the type and rate of HCW. At the outset of the comprehensive fieldwork, a reconnaissance survey was carried out to identify the overall management status and physical composition of HCW in the study area. Due to time constraints, primary data were collected from selected HCEs during July to August 2015, so as to quantify the generation of waste. Congregated wastes were then segregated and characterized following its classification and finally weighted in the separate room. Prüss et al. [23] classified HCW in nine categories (infectious, sharps, pathological, pharmaceutical, genotoxic, and chemical waste, waste with high content of heavy metals, and pressurized containers and radioactive waste). However, the reconnaissance survey unveiled that all these wastes were not available in the study area or produced in a very insignificant amount which have not been included in the present investigation. In this study, the infectious, pathological, chemical, sharps, and plastic-type waste have been considered as hazardous waste and general waste as nonhazardous.

Waste sampling was performed once per day and the quantity of the HCW generated was then recorded according to its classification. Questionnaire surveys and in-depth interviews were adopted to acquire qualitative information. Questionnaires were designed from studying previous research on this topic, so that they would cover all basic requirements needed for HCWM in Khulna city. It is noted here that four (4) sets of questionnaires were prepared for different categories of respondents including patients, doctors, nurses, technicians, cleaners, administrative officers, and management authorities. Collected data were then analyzed to yield findings (see Section 5.2). Hazardous waste treatment alternatives were assessed using Sustainability Assessment of Technologies (SAT) methodology. The SAT outcomes, therefore, assisted in informative decision-making for choosing the most suitable waste treatment technology. Secondary data were gathered from various public and private organizations, different workers, officials, some journals, publications, websites, and so on.

### 4.3. Employing SAT Methodology

In the strategic level, the present status of HCWM in the Khulna metropolitan area was analyzed involving the collection of baseline data and stakeholder consultations. Then, the target was set to manage the HCW efficiently using suitable treatment technology. Afterward, the methodology was moved on to an operational level assessment where common incineration, microwave, autoclave, waste treatment alternatives, and so on were screened by using modified screening criteria through consultative meetings to meet local situations. Waste treatment technologies that passed through the screening step were then subjected to the comprehensive scoping tier. Scoping criteria were developed under four broad criteria topics as technical, financial, social, and environmental. It is noted here that there were 10 technical, 18 environmental, 4 financial, and 5 social criteria under each criteria topic. Weight was given by the stakeholders for each criterion from 0 to 10 depending on the importance and significance (0 for not important, 1 to 3 for low, 4 to 6 for medium, 7 to 9 for high, and 10 for essential). For each waste treatment technology under consideration, the score was given for each criterion from 0 to 9 (1 to 3 for low, 4 to 6 for medium, and 7 to 9 for high). For each criterion multiplying factors were calculated as follows [12]:

(1) MF=W×RCTMSCT,MSCT=9×algebraic  sum  of  all  weightage  given  under  each  criteria  topic,

where *W* is weight of each criterion, RCT is ranking for each criteria topic, and MSCT is maximum score for each criteria topic.

It can be mentioned here that the ranking of each criteria topic (RCT) is established by a factor from 0 to 100 to each topic such that the sum of all the ranking factors adds up to 100. In this study, 25 was assigned to each topic since they are all of equal importance according to the local conditions. The technologies with best overall ratings from the scoping step were selected for further assessment in this step. Among various multicriteria decision-making (MCDM), the simple weighted sum matrix method was employed in this study for quantitative assessment. With the aim of selecting the most preferred HCW treatment technology, a number of experts and stakeholders in the study area participated in the SAT process. For detailed assessment outcomes, readers are suggested to see Section 5.5.

## 5. Results and Discussions

### 5.1. Generation of HCW in the Studied HCEs

The study revealed that waste generation rate is slightly higher (*p* < 0.05) in Public HCEs (0.95 kg bed^−1^ day^−1^) than the private health centers (0.88 kg bed^−1^ day^−1^). Public HCFs produced more wastes than that of the Private HCEs due to more numbers of beds, departments, and wards in comparison with private hospitals [24]. The amount of HCW generated in the HCEs was positively correlated with the number of beds (*r*_s_ = 0.79, *p* < 0.001). The average waste generation per bed per day in Khulna city has been found to be 0.90 kg (Table 1), which is much lower than that of the developed countries like the United States (4.5 kg bed^−1^ day^−1^), the United Kingdom (3.3 kg bed^−1^ day^−1^), and Spain (4.4 kg bed^−1^ day^−1^) [25, 26]. In high-income countries, HCW generation is usually higher than that in the middle and low-income countries [27]. The rate of waste generation mainly depends upon geographical location, living standard, healthcare facilities, waste collection services, and so on. In Latin American countries like Chile, Brazil, Argentina, and Venezuela, this figure varies from 1 to 4.5 kg bed^−1^ day^−1^ [28]. Therefore, it can be mentioned that the waste generation rate of Khulna city is close to the figure of the developing countries of Latin America. However, this figure is very close to the generation rate of 0.93 kg bed^−1^ day^−1^ in Sylhet city and 1.2 kg bed^−1^ day^−1^ in Dhaka [29, 30].

**Figure 1:**
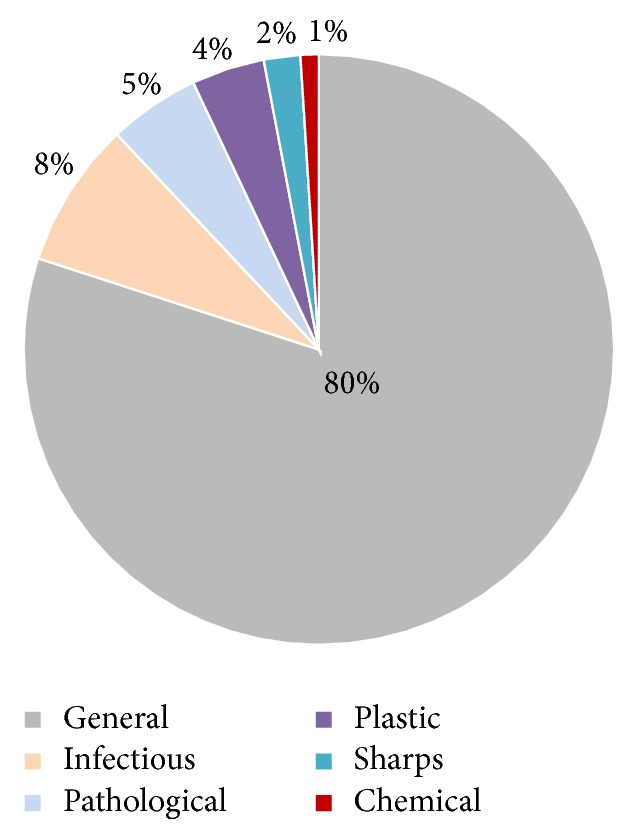
Composition of HCW in Khulna city.

The rate of generation of hazardous waste per bed per day from selected HCEs in Khulna city has been found to be 0.18 kg (Table 1). This rate is close to the rate of 0.21 kg bed^−1^ day^−1^ in Sylhet and Bangladesh [29] and much lower than that of 0.57 kg bed^−1^ day^−1^ in Brazil and 0.47 kg bed^−1^ day^−1^ in Japan reported by Da Silva et al. [31] and Mohee [32], respectively. About 80% of waste generated is nonhazardous and the other 20% is hazardous (Figure 1) which is much lower than reported in Denmark (25%) and the United States (28%) [33]. Among hazardous waste, 8% is infectious, 5% pathological, 4% plastic, 2% sharps, and 1% is chemical waste (Figure 1).

About 3 tons of HCW were generated daily from 120 registered HCEs in Khulna city (Table 2). Most of them were contributed by private HCFs (60%) as the aggregated number of beds is higher in Private HCEs. Public HCFs cover 35% and the other 5% were generated by the diagnostic centers and dental clinics. In Khulna city, about 235 kg of infectious waste, 116 kg of plastic, 24 kg of chemicals, and 69 kg of sharps are generated daily from different HCEs (Table 2). These large amounts of unorthodox wastes deserved extra attention.

The importance for the proper management of HCW can be sensed from the aforementioned statistics which revealed that roughly 212 metric tons of hazardous waste is generated in Khulna each year. This ever-increasing amount of hazardous waste, if not managed properly will cause severe health hazards and environmental problems.

### 5.2. Survey Findings Depicting HCWM Practices in Khulna City

The materials presented here are aimed at showing respondents view on the existing HCWM practices in different HCEs in the KCC area. Using a random sampling procedure, a questionnaire survey was carried out among 87 respondents from different occupations including patients, doctors, nurses, cleaners, administrators, and general people in different HCFs. Most of them were educated, with the age ranging from 30 to 50 years and average service length of 10 years. However, a few of them were illiterate. Their opinions are displayed herein (Table 3).

In the studied HCEs, the frequency of on-site waste handling was categorized as once per day, twice per day, and irregular. Most of the respondents in Private HCEs opine that the on-site waste handling (patient's bed to storage place) is irregular while in Public HCEs, most of them said that it is once per day. In the case of on-site waste handling from storage place to municipal dustbin or NGOs van, the majority from the Private HCEs reported on once per day which can be evident from Table 3. More than 40 respondents reported that waste collection from the secondary source to final disposal was at noon while a good number of respondents enunciated on morning.

In the case of an existing level of awareness on using safety equipment to prevent the spreading of infectious diseases, a great number of respondents (54%, *n* = 47) from HCEs reported that they were not using any safety equipment (Table 3). It also investigated from the field survey that almost all the respondents from surveyed HCEs focused their opinion in favor of training concerning the waste management. In connection with the training methods, the mainstream showed their interest in video and lecture.

**Figure 2:**
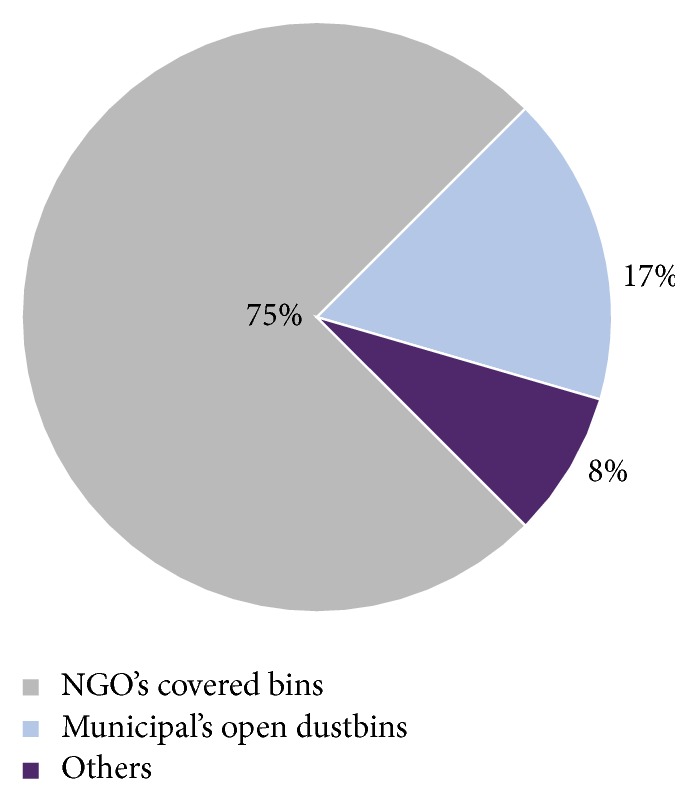
Graphical presentation of preferable waste collection system.

It was observed from the study that 75% (*n* = 65) of the total respondents preferred to dispose of their wastes in the NGO's covered bins. 17% (*n* = 15) of respondents indicated the municipal open dustbin for their waste disposal and very few respondents reported on another disposal system (Figure 2).

A large number of respondents (58%, *n* = 50) expressed their dissatisfaction with existing HCWM practices in Khulna city. The problems they faced are no environment-friendly dustbin for waste disposal, no separate dustbin for HCW collection, dustbin is not in a suitable location, waste collection system is unhygienic, irregular collection system, and hazardous waste is spreading all over the place due to lack of on-site treatment facility. Although the chart shows that satisfaction rates are quite high, this is due to the better management of Private HCEs. They also mentioned some probable steps that could overcome the problems and these are as follows:

- Use of an apron, mask, and gloves during handling the patients and segregation as well as disposal of wastes; use of WHO-guided color-coded bins for segregated waste;
- Training for awareness could be a great help regarding this issue;
- Formulating, amending, and imposing the relevant laws could prevent the improper management of HCW.

### 5.3. Institutional Arrangements for HCWM in Khulna City and Their Limitations

At the local level, only KCC, a public organization, and Prodipan, an NGO, are involved in HCWM in Khulna city. KCC does not have any special arrangement for the collection and disposal of HCW separately as they are not obliged to do according to their ordinance. The conservancy department of KCC identifies the lack of trained manpower and resources as the principal reasons for not having any arrangement to handle the HCW. KCC does not have any research facility to identify the extent of different problems and to provide guidelines to mitigate that. Around 47% of the money spent on waste management goes for the maintenance purpose of the vehicle and the remaining 53% is spent on providing remuneration of the staff engaged in solid waste management in KCC. KCC collected the HCW in the same vehicle together with other wastes from the public dustbin twice a day and dumped it together in the dumping ground near Rajbandh, about 7 km south of the city. KCC also dumped wastes for landfilling purpose at different locations of the city. It increased the risk of health hazards to the adjacent community.

On the other hand, Prodipan started their journey in May 2000, with the financial aid from the Swiss Development Cooperation, UNDP, and the World Bank. Initially, they started their project with 20 HCEs, which increased to 100 in 2013. Prodipan segregated wastes at the source of segregation. They provided a set of four covered drums to dispose of four types of waste separately. An autocovered van of 1.0-ton capacity was used for transportation of HCW from different HCEs. Generally, collection took place in the morning every day. Prodipan took service charge from the HCEs they served and the charge was determined depending on the size (number of bed) and earnings of the HCEs. The study revealed that 44% of monthly expenditure is spent on providing wages for the staff. Vehicle maintenance cost is 46% and the remaining 10% is for purchasing the required materials. Prodipan burnt infectious waste in a locally made burning pit at a comparatively low temperature (about 400°C or below). It may, in some cases, cause unfinished burning and in the case of the presence of any type of plastic material in the waste, experts opine that “this is more harmful as it helps in producing dioxin gas.” They disposed of the needles and all other sharp materials in a concrete pit. This could be viable at a small scale; however, in the case of the entire city, where the yearly generation of sharps waste is about 25 metric tons, the volume reduction is crucial. The present HCWM system of Prodipan is not a very structured and cost-effective one. The project is not internally balanced and continuity of the project is totally dependent on the availability of foreign aid.

### 5.4. Existing HCWM Practices in Khulna City and Its Impacts

With the upward trend of population and mushrooming growth of HCEs together with lack of operational waste disposal mechanism, the environmental condition of Khulna city is gradually becoming more alarming. The existing HCWM status in the KCC area is unsatisfactory and unsafe for health. Most of the HCEs in Khulna had no apposite waste management system and they did not use any sort of protective clothing like gloves, a mask, and so on. Hospital authorities were found to be less concerned regarding proper disposal of clinical waste. Although proper segregation and treatment of infectious waste before dumping are very crucial to minimizing health risks to the community [34], they had not been exercised in any of the HCEs studied. It was revealed from the study that more than 80% of HCW is nonhazardous which may be considered as general waste. This huge amount of nonhazardous waste is excessively contaminated with hazardous waste due to a lack of proper waste separation practices. Moreover, there are no distinct color-coded collection bins for HCWM and all categories of waste from HCEs comprising reusable and sharp waste are dumped in common places like public garbage disposal bins, side of roads, dumping grounds, or municipal waste collection containers. This malpractice elucidated the inefficiency of HCWM in Khulna as well as increasing the chances of contamination of an entire mass of solid waste tainting it with infectious HCW.

HCW comprises biodegradable and nonbiodegradable polymers [29]. Biodegradable polymers are easily decomposed by the action of microorganisms, while nonbiodegradables are very difficult to decay. The change in biological character of HCW disinfects it, which reduces the infectious biohazardous properties of the waste [35]. The microorganisms may create a cyst to stay alive in adverse condition and contaminate the environment [29]. Chemical effluents produced from several HCEs were released straight into the municipal cesspool and may have toxic effects on the natural ecosystems of receiving waters. Most landfills are not constructed properly, which may contaminate drinking water. Surface overflow directly from deposited waste can pollute surface water easily. Direct ejection of blood, body parts, feces, and urine of contagious patients in a public sewer system may cause a spate of communicable diseases. Lack of awareness regarding the damaging effects of HCW was also found among the workers involved in the total management process as well as general people in the studied area.

Most of the disposal site is open and thereby emits unpleasant odors and an unaesthetic view, causing a huge public nuisance. While grazing, ready access of domestic animals in open dumps may create the possibilities of introducing microbes and pathogens into the food chain. Indiscriminate junking of HCW may create the chances of adulteration of food supplies, soil, surface water, groundwater, and air. The majority of the municipal waste receptacles are not designed appropriately and are open without a cover or lid. Therefore, vectors, like insects, rodents, worms, birds, and so on, can easily enter the collection containers and can take a place on the exposed piles of rotting trash causing the spread of contagious bugs. These also stimulate the mechanical transmission of deadly waterborne diseases like diarrhea, typhoid, dysentery, hepatitis, and cholera [29]. Moreover, mosquitoes promote biological transmission of many types of diseases like malaria, dengue, and yellow fever under humid environment. Rubber and plastic trash being burnt in the open air releases fumes containing carbon monoxide, dioxins, furans, and so on [36]. When these toxic components are inhaled through smoke, they may cause cancer, respiratory diseases, and many other deadly results to humans.

Informal waste collectors (known as scavengers) engaged in collecting refuse from HCEs are suffering from various intestinal, parasitic, and skin diseases. Waste pickers collect used medical equipment, particularly syringes, from the garbage and sell them at a low price. Many drug addicts may suffer from cholera, typhoid, hepatitis, AIDS, and other hazardous and contagious diseases as they are reusing these syringes. Scavengers are scooping out waste from the dustbins, roads, and garbage lots for the recyclables with bare hands, without taking any safety measures, therefore, facing a high risk of salient epidemics of infectious diseases. The waste pickers involved in the recycling process are extremely poor, having no proper education, and incautious of detrimental consequences of exposure to contaminated and harmful waste.

The inadequate disposal of HCW may be catastrophic to health and the environment as well as the wellbeing of society. If the HCW in Khulna city is not handled in a proper way, it will undoubtedly pose a danger to the workforce employed in the HCEs as well as to the neighboring people.

### 5.5. Assessment of Different Technological Options for Proper Treatment of HCW Using SAT Methodology in the Context of Khulna City

In general, treatment options may be available either on- or off-site. A central treatment facility is proposed as the generation of hazardous waste from individual HCEs is not high enough to run a treatment facility solely. Assessment outcomes of different technological choices for HCW treatment including technical suitability, environmental, economic, and social aspects using SAT methodology for Khulna are narrated herein chronologically.

*Strategic-Level Assessment*

- Issue: all HCEs in the Khulna metropolitan area do not have proper treatment facilities to treat their hazardous waste efficiently.
- Target: implementation of a large-scale central treatment technology to handle the hazardous waste from all HCEs in the area. Furthermore, proclamation of policies to be needed for the treatment of all hazardous waste.

*Operational Level Assessment. *Various HCW treatment alternatives, for instance, incineration, microwaving, and autoclaving were screened (Table 4) in this step using different modified screening criteria. Short-listed technological options passed through the screening step were then subjected to going through the comprehensive scoping tier (Table 5).

**Figure 3:**
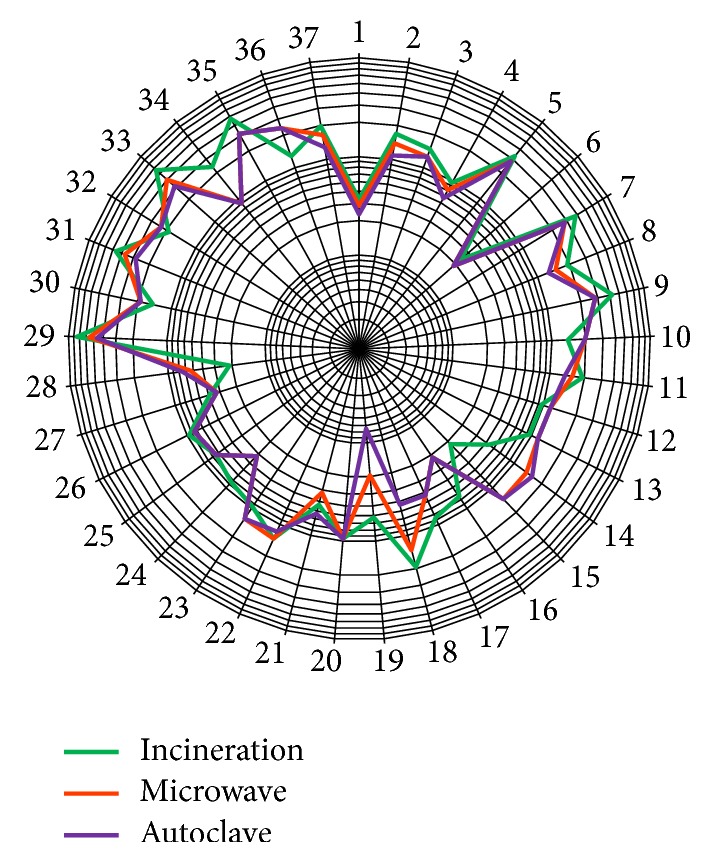
Distribution of acquired scores of all criteria of HCW treatment options.

*Detailed Assessment. *The final scores obtained by different treatment alternatives for the technical suitability and economic, social, and environmental criteria are shown in Table 6. Final weightage of all criteria (37 numbers) under each criteria topic for HCW treatment options is presented in a composite star diagram (Figure 3).

It is observed from Table 6 that all three technologies are fairly close although incineration is the highest ranked treatment option followed by microwave technology. Among different criteria, technical suitability and social aspect vary to some extent. In the technical suitability aspect, the most important criteria considered by the experts judgment were the ability to treat a wide range of hazardous wastes, capacity requirement, and adaptability to the future situation. Regarding the social aspect, experts contemplated community acceptance of the technology. Although incineration attained the highest score in technical suitability and social aspect, the capital cost of the treatment technology as well as maintenance expenditure is one of the drawbacks of this treatment option. Özkan [22] indicated that off-site incineration is the best method out of the five assessed HCW treatment alternatives. Another research using the multicriteria decision-making (MCDM) approach carried out by Antonopoulos et al. [37] also corroborated the same facts.

Based on assessment outcomes utilizing SAT methodology, characterization of waste, and local conditions, incineration is likely said to be the suitable method for treatment of all types of HCW in Khulna city. The majority of the wastes produced from HCEs are nonhazardous which can be disposed of in ways similar to the domestic waste. Only the hazardous portion needs to be treated. In Khulna city, the present generation of the waste is about 3 ton day^−1^ and the hazardous waste is nearly 0.6 ton day^−1^. Therefore, a medium size incinerator (destruction capacity: 0.25 ton hr^−1^) will be good enough to comply with the situation. Unwanted pollutants can be significantly reduced with the addition of air pollution control devices of the system. Many air pollutants in emissions from incinerators can be lessened substantially by modern air pollution control mechanisms if appropriately designed and operated [2, 38]. Since the investment cost is very high for new plant, several HCEs may converge to set up a plant centrally. These types of plants are now being observed in many countries of the world.

## 6. Conclusions and Recommendations

The present study has mainly focused on the existing HCWM paradigms of Khulna metropolis and on the question of how it can be made a more efficient and acceptable one. The existing HCWM pattern in Khulna city has many drawbacks and is in dire need of immediate attention and improvement. It was observed from the investigation that the hazardous waste was not treated separately in almost all of the HCEs in Khulna city. Intervention is required at all stages of waste management from the formulation of appropriate laws, segregation, and transportation of waste to the final disposal method. The process and method adopted for waste management should be technically and financially sustainable in the long run. It has to also be ensured that there are no adverse health and environmental consequences of waste handling, treatment, and disposal activities.

National legislation is the basis for improving HCW practices in any country. Therefore, a national management plan will be required which will permit HCWM options to be optimized on a national scale. The law should be complemented by a policy document and technical guidelines developed for implementation. This legal document should specify regulations on treatment for different waste categories, segregation, collection, storage, handling, disposal, transportation, responsibilities, and training requirements. Training of healthcare personnel as well as general people regarding hygiene and HCWM is needed to create awareness and foster responsibility among them which will prevent exposure to related health hazards.

Among different waste treatment options, it has been found from the study that the incineration system is the most suitable one for Khulna city based on the final score considering technical suitability and environmental, economic, and social aspect. However, the system should be maintained properly with an appropriate air pollution control device. The ranking order of the second technological choice was microwaving followed by autoclaving, considering all aspects. The assessment of treatment alternatives in this investigation is subjected to the selection as well as weighting of the criteria and strongly dependent on the reliability to the response of the experts' personal judgment. Besides, the waste generation rate that was calculated in this investigation was excluding seasonal variation. A further detailed study is required incorporating more HCEs with an extended period of collected data as well as seasonal variation to explore the sustainability of such management option. Furthermore, it is recommended to develop a great variety of MCDM methods to evaluate such HCW treatment alternatives. Overall, the study will give an insight promulgating guidelines for the future planning and design of HCWM strategies in Khulna city as well as other municipalities in developing countries.

## Figures and Tables

**Table 1:** Overall HCW (hazardous and nonhazardous) generation rate from different HCEs in Khulna city.

Name of the HCE	Number of beds	Category	Total generated waste, kg bed^−1^ day^−1^ or kg DC^−1^ day^−1^	Mean, kg bed^−1^ day^−1^ or kg DC^−1^ day^−1^	Haz. waste, kg bed^−1^ day^−1^ or kg DC^−1^ day^−1^	Mean, kg bed^−1^ day^−1^ or kg DC^−1^ day^−1^
KGH	250	Public	0.98	0.90	0.20	0.18
KMCH	500	Public	1.15		0.25	
SANSH	75	Public	0.89		0.19	
IDH	20	Public	0.87		0.17	
CDH	100	Public	0.90		0.19	
MCWC	20	Public	0.88		0.17	
GMCH	500	Private	1.00		0.20	
GNCDC	50	Private	0.88		0.15	
AAMCH	20	Private	0.80		0.17	
KSH	218	Private	0.92		0.19	
KDH	100	Private	0.90		0.18	
KSMH	50	Private	0.87		0.16	
DBC	30	Private	0.85		0.15	
IBH	100	Private	0.91		0.17	
FHC	15	Private	0.77		0.12	
KC	25	Private	0.83		0.14	

DDC	--	DC	3.10	4.42	1.19	1.71
MDC	--	DC	5.90		2.11	
SDC	--	DC	4.50		1.80	
PDC	--	DC	4.20		1.74	

*Notes*. DC: Diagnostic Center or Dental Clinic, KGH: Khulna General Hospital, KMCH: Khulna Medical College Hospital, SANSH: Sheikh Abu Naser Specialized Hospital, IDH: Infectious Diseases Hospital; CDH: Cheast Disease Hospital, MCWC: Mother & Child Welfare Center, GMCH: Gazi Medical College Hospital, GNCDC: Garib Newaz Clinic and Diagnostic Center, AAMCH: Ad-din Akij Medical College Hospital, KSH: Khulna Shishu Hospital, KDH: Khulna Diabetic Hospital, KSMH: Khulna Surgical and Medical Hospital, DBC: Dabs Clinic, IBH: Islami Bank Hospital, FHC: Fair Health Clinic, KC: Khalishpur Clinic, DDC: Decent Dental Clinic, MDC: Mahanagar Diagnostic Center, SDC: Setu Diagnostic Center, and PDC: Padma Diagnostic Center.

**Table 2:** Total amount of HCW generated in Khulna city.

Types of HCEs	Nonhazardous waste (Kg day^−1^)	Infectious waste (Kg day^−1^)	Pathological waste (Kg day^−1^)	Chemical waste (Kg day^−1^)	Plastic waste (Kg day^−1^)	Sharp waste (Kg day^−1^)	Total (Kg day^−1^)
Public	793	86	47	8	41	23	998
Private	1399	135	83	11	56	32	1716
DC	96	14	7	5	19	14	155
Grand Total	2288	235	137	24	116	69	2869

*Note*. This figure was calculated only from 120 registered HCEs (Total number of beds: 3000). It can be mentioned that there were more than 50 nonregistered HCEs existing in Khulna city. These HCFs produced relatively small portion of the overall amount of waste in the studied area.

**Table 3:** Scenarios of waste handling and management practices in Khulna metropolis.

Variables	Frequency (%)			Total respondents (%)
	GH	PH	DC	
*On-site handling (patient's bed to storage place)*				
Once per day	12 (14)	10 (11)	4 (5)	30
Twice per day	4 (5)	13 (15)	5 (6)	25
Irregular	8 (9)	19 (22)	12 (14)	45
*On-site handling (storage place to final disposal)*				
Once per day	14 (16)	34 (39)	15 (17)	73
Twice per day	6 (7)	5 (6)	4 (5)	17
Irregular	4 (5)	3 (3)	2 (2)	10
*Time of waste collection (secondary source to final disposal)*				
Morning	10 (12)	18 (21)	4 (5)	37
Noon	24 (28)	14 (16)	5 (6)	49
Random	9 (10)	2 (2)	1 (1)	14
*Systematic issues of waste collection*				
Systematic	21 (24)	7 (8)	5 (6)	38
Nonsystematic	3 (3)	35 (40)	16 (18)	62
*Safety equipment scenario*				
Followed	7 (8)	23 (26)	10 (12)	46
Not followed	17 (20)	18 (21)	12 (14)	54
*Training issues*				
Got training	5 (7)	19 (28)	6 (9)	44
Did not get training	14 (21)	15 (22)	9 (13)	56
*Opinion on training methodology*				
Lecture & video	16 (24)	28 (41)	12 (18)	82
Video	2 (3)	3 (4)	2 (3)	10
Lecture	1 (1)	3 (4)	1 (1)	7
*Opinion on existing HCWM*				
Satisfactory	2 (2)	25 (29)	10 (11)	42
Unsatisfactory	25 (29)	16 (18)	9 (10)	58

*Notes*. GH: public HCEs, PH: private HCEs, DC: diagnostic center or dental clinic.

**Table 4:** Screening of different HCW treatment alternatives for Khulna municipality.

Criteria	Incineration with pollution control device	Microwave	Autoclave	Hydropulping	Compaction
Compliance with national environmental laws	√	√	√	X	X
Compliance with multilateral environmental agreements	√	√	√	√	√
Consistency with WHO policies	√	√	√	√	√
Flexibility in accepting various waste	√	√	√	X	X
Volume reduction	√	√	X	√	√
Technology economically Viable	√	√	√	√	√
Acceptability of technology in Khulna	√	√	√	X	X
Outcome (selected/not selected)	√	√	√	X	X

*Note*. √: yes and X: no.

**Table 5:** Scoping of SAT methodology for the assessment of HCW treatment options for Khulna metropolis.

Criteria	Weight (*W*)	Ranking for criteria topic (RCT)	Maximum score for criteria topic (MSCT)	Multiplying Factors (MF) = *W* × RCT/MSCT	Incinerator		Microwave		Autoclave	
					Score	Score × MF	Score	Score × MF	Score	Score × MF
*Technical suitability *										
Compatibility with local surroundings and natural conditions	*W*1 = 1	Ranking for Technical Suitability, RT: 25	Maximum score for Technical Suitability (MST): 9 × (*W*1 + *W*2 + ⋯+*W*10) = 531	MF1 = 0.047	7	0.329	6	0.282	5	0.235
Preference for locally manufactured technologies	*W*2 = 7			MF2 = 0.329	5	1.645	4	1.316	3	0.987
Availability of spare parts and usage of local materials	*W*3 = 5			MF3 = 0.235	6	1.410	5	1.175	5	1.175
Availability of local expertise	*W*4 = 3			MF4 = 0.141	6	0.846	5	0.705	4	0.564
Track record on performance	*W*5 = 10			MF5 = 0.470	7	3.290	6	2.820	6	2.820
Compatibility with existing technology or management system	*W*6 = 1			MF6 = 0.047	5	0.235	4	0.188	4	0.188
Meets capacity requirement	*W*7 = 10			MF7 = 0.470	8	3.760	6	2.820	6	2.820
Adaptability to future situations	*W*8 = 5			MF8 = 0.235	8	1.880	6	1.410	5	1.175
Ability to treat a wide the range of healthcare wastes	*W*9 = 10			MF9 = 0.470	9	4.230	6	2.820	6	2.820
Level of automation/sophistication	*W*10 = 7			MF10 = 0.329	4	1.316	6	1.974	6	1.974

*Environment*										
Efficacy of microbial inactivation	*W*11 = 10	Ranking for environment (resources and emissions), REn: 25	Maximum score for environment (MSEn): 9 × (*W*11 + *W*12 + ⋯+*W*28) = 1161	MF11 = 0.215	9	1.935	7	1.505	6	1.290
Risk levels for workers	*W*12 = 10			MF12 = 0.215	4	0.860	5	1.075	5	1.075
Risk levels for communities	*W*13 = 10			MF13 = 0.215	4	0.860	5	1.075	5	1.075
Risk to the environment	*W*14 = 10			MF14 = 0.215	2	0.430	6	1.290	7	1.505
Air emissions	*W*15 = 10			MF15 = 0.215	1	0.215	6	1.290	6	1.290
Liquid effluents	*W*16 = 5			MF16 = 0.107	6	0.642	2	0.214	2	0.214
Solid residues	*W*17 = 5			MF17 = 0.107	7	0.749	4	0.428	4	0.428
Volume reduction	*W*18 = 10			MF18 = 0.215	9	1.935	6	1.290	2	0.430
Mass reduction	*W*19 = 3			MF19 = 0.065	8	0.520	3	0.195	1	0.065
Odor	*W*20 = 10			MF20 = 0.215	4	0.860	4	0.860	4	0.860
Noise	*W*21 = 5			MF21 = 0.107	4	0.428	3	0.321	5	0.535
Energy consumption per kg of waste	*W*22 = 10			MF22 = 0.215	6	1.290	6	1.290	5	1.075
Extent of use of renewable energy	*W*23 = 8			MF23 = 0.172	5	0.860	7	1.204	7	1.204
Water consumption per kg of waste	*W*24 = 5			MF24 = 0.107	7	0.749	3	0.321	3	0.321
Material consumption	*W*25 = 5			MF25 = 0.107	6	0.642	6	0.642	6	0.642
Extent of use of hazardous materials	*W*26 = 5			MF26 = 0.107	8	0.865	7	0.749	7	0.749
Space requirement	*W*27 = 3			MF27 = 0.065	6	0.390	5	0.325	5	0.325
Resource recovery capabilities	*W*28 = 5			MF28 = 0.107	2	0.214	5	0.535	6	0.642

*Financial/economical*										
Estimated capital cost of the treatment technology	*W*29 = 10	Ranking for economic aspects, REc: 25	Maximum score for economic (MSEn): 9 × (*W*29 + *W*30 + ⋯+*W*32) = 270	MF29 = 0.926	8	7.408	6	5.556	5	4.630
Estimated capital costs of all accessories and related equipment	*W*30 = 5			MF30 = 0.463	3	1.389	4	1.852	4	1.852
Estimated operation and maintenance costs	*W*31 = 10			MF31 = 0.926	5	4.630	4	3.704	3	2.778
Installation requirements	*W*32 = 5			MF32 = 0.463	4	1.852	5	2.315	5	2.315

*Social/cultural*										
Community acceptance of the technology	*W*33 = 10	Ranking for social aspects, RS: 25	Maximum score for Social/Cultural (MSS): 9 × (*W*33 + *W*34 + ⋯+*W*37) = 315	MF33 = 0.794	7	5.558	5	3.970	4	3.176
Income generation potential	*W*34 = 5			MF34 = 0.397	6	2.382	2	0.794	2	0.794
Acceptability of treatment residues by the local landfill	*W*35 = 10			MF35 = 0.794	6	4.764	4	3.176	4	3.176
Extent of necessary resettlement of people	*W*36 = 5			MF36 = 0.397	3	1.191	6	2.382	6	2.382
Visible or aesthetic impact	*W*37 = 5			MF37 = 0.397	5	1.985	4	1.588	3	1.191

*Total*						64.5		55.4		51.2

**Table 6:** Ranking of different HCW treatment options based on final scores.

Technological options	Technical suitability	Environmental	Economic	Social	Total	Rank
Incineration	18.9	14.4	15.3	15.9	64.5	1
Microwave	15.5	14.6	13.4	11.9	55.4	2
Autoclave	14.8	13.7	11.9	10.7	51.2	3

## Abbreviations

HCEs: Healthcare establishments
HCFs: Healthcare facilities
HCW: Healthcare waste
HCWM: Healthcare waste management
IETC: International Environmental Technology Centre
KCC: Khulna City Corporation
MCDM: Multicriteria decision-making
NGO: Nongovernmental organization
SAT: Sustainability Assessment of Technology
UNDP: United Nations Development Program
UNEP: United Nations Environment Program
WHO: World Health Organization.
